# Comprehensive analysis of disulfidptosis-related genes: a prognosis model construction and tumor microenvironment characterization in clear cell renal cell carcinoma

**DOI:** 10.18632/aging.205550

**Published:** 2024-02-14

**Authors:** Bocun Yi, Xifeng Wei, Dongze Liu, Liwei Jing, Shengxian Xu, Man Zhang, Zhengxin Liang, Ranlu Liu, Zhihong Zhang

**Affiliations:** 1 Department of Urology, Tianjin Institute of Urology, The Second Hospital of Tianjin Medical University, Tianjin, China; 2 Department of Urology, People’s Hospital of Ningxia Hui Autonomous Region, Yinchuan, China; 3 Tianjin Key Laboratory of Metabolic Diseases, Tianjin Institute of Endocrinology, Chu Hsien-I Memorial Hospital of Tianjin Medical University, Tianjin, China

**Keywords:** disulfidptosis, clear cell renal cell carcinoma, cancer subtypes, prognosis model, tumor immune microenvironment

## Abstract

Background: Disulfidptosis, a form of cell death induced by abnormal intracellular accumulation of disulfides, is a newly recognized variety of cell death. Clear cell renal cell carcinoma (ccRCC) is a usual urological tumor that poses serious health risks. There are few studies of disulfidptosis-related genes (DRGs) in ccRCC so far.

Methods: The expression, transcriptional variants, and prognostic role of DRGs were assessed. Based on DRGs, consensus unsupervised clustering analysis was performed to stratify ccRCC patients into various subtypes and constructed a DRG risk scoring model. Patients were stratified into high or low-risk groups by this model. We focused on assessing the discrepancy in prognosis, TME, chemotherapeutic susceptibility, and landscape of immune between the two risk groups. Finally, we validated the expression and explored the biological function of the risk scoring gene FLRT3 through *in vitro* experiments.

Results: The different subtypes had significantly different gene expression, immune, and prognostic landscapes. In the two risk groups, the high-risk group had higher TME scores, more significant immune cell infiltration, and a higher probability of benefiting from immunotherapy, but had a worse prognosis. There were also remarkable differences in chemotherapeutic susceptibility between the two risk groups. In ccRCC cells, the expression of FLRT3 was shown to be lower and its overexpression caused a decrease in cell proliferation and metastatic capacity.

Conclusions: Starting from disulfidptosis, we established a new risk scoring model which can provide new ideas for doctors to forecast patient survival and determine clinical treatment plans.

## INTRODUCTION

Renal cell carcinoma (RCC) is one of the 10 most usual types of cancer [1]. Clear cell renal cell carcinoma (ccRCC), also named kidney renal clear cell carcinoma (KIRC), occupies about 70–80% of RCC cases, posing a serious threat to human health [2]. Early ccRCC can be treated well with surgery and other treatments, but one-third of patients diagnosed with ccRCC have metastases from the primary lesion [3]. Although chemotherapy and immunotherapy are also available, the heterogeneity of ccRCC makes the treatment outcome of patients various [1, 4]. In addition, simply using TMN staging is not an accurate predictor of patient survival [5]. Therefore, it is necessary to research and develop new biomarkers for prognostic prediction and therapeutic targeting in ccRCC.

Disulfidptosis is a newly characterized form of cell death. In brief, the abnormal accumulation of intracellular disulfides in cells with high solute carrier family 7 member 11 (SLC7A11) expression under glucose starvation leads to cell death [6]. Under glucose starvation conditions, high expression of SLC7A11 in renal cancer cells accelerates the depletion of nicotinamide adenine dinucleotide phosphate in the cytoplasm. This leads to the accumulation of irreducible disulfides, inducing disulfide stress and ultimately disulfide death. SLC7A11, overexpressed in various human cancers, is the cystine/glutamate reverse transporter protein that was used to import cystine for glutathione biosynthesis and antioxidant defense, it is responsible for cystine uptake, and its high expression in renal cancer cells mediates the high rate of cystine uptake. Accumulation of disulfides such as cystine induces disulfide stress, which is toxic to cells [7–10]. But previous research related to SLC7A11 has mainly discussed its role in cellular ferroptosis. Moreover, previous studies have demonstrated that disulfides are toxic to cancer cells and could be a potential anticancer treatment [11, 12]. However, there are few studies on the link between the newly defined disulfidptosis and the occurrence, tumor microenvironment, or treatment of ccRCC. Therefore, it is necessary to explore whether disulfidptosis plays a key role in ccRCC.

In this research, we identified ten genes (GYS1, LRPPRC, NCKAP1, NDUFA11, NDUFS1, NUBPL, OXSM, RPN1, SLC3A2, and SLC7A11) that are closely related to disulfidptosis as disulfidptosis-related genes (DRGs) and investigated them in a multi-omics approach to comprehensively analyze their role in ccRCC. The ultimate goal is to construct a new prognostic model for predicting the prognosis and guiding the treatment of patients with ccRCC. First, ccRCC patients were classified into three DRG-related molecular subtypes according to the expression levels of DRGs. The prognosis-related differentially expressed genes in these three clusters were then used to classify patients into two DRG-related genetic subtypes. We discussed the gene expression, immune, and prognostic landscape between the different molecular subtypes or genetic subtypes. Then, we pinpointed 5 risk scoring genes (FLRT3, ATP1A1, SAA1, PDK4, and KCNJ15) among the prognosis-related DEGs and constructed a novel DRG-related risk scoring model for ccRCC patients. In the testing group and E-MTAB-1980 cohort, we validated the stability and precision of the risk scoring model. Additionally, we analyzed the relationship between risk score and prognosis, immune landscape, mutation, TMB, chemotherapy, and immunotherapy of ccRCC patients to assess the role of the DRG risk scoring model in molecular therapy in more depth. Finally, FLRT3 was chosen as an important biomarker and *in vitro* experiments were conducted to verify its expression in normal and ccRCC cells as well as to investigate its effect on the biological behavior of ccRCC.

## RESULTS

### Expression and genetic alteration of DRGs in ccRCC

**Figure 1:**
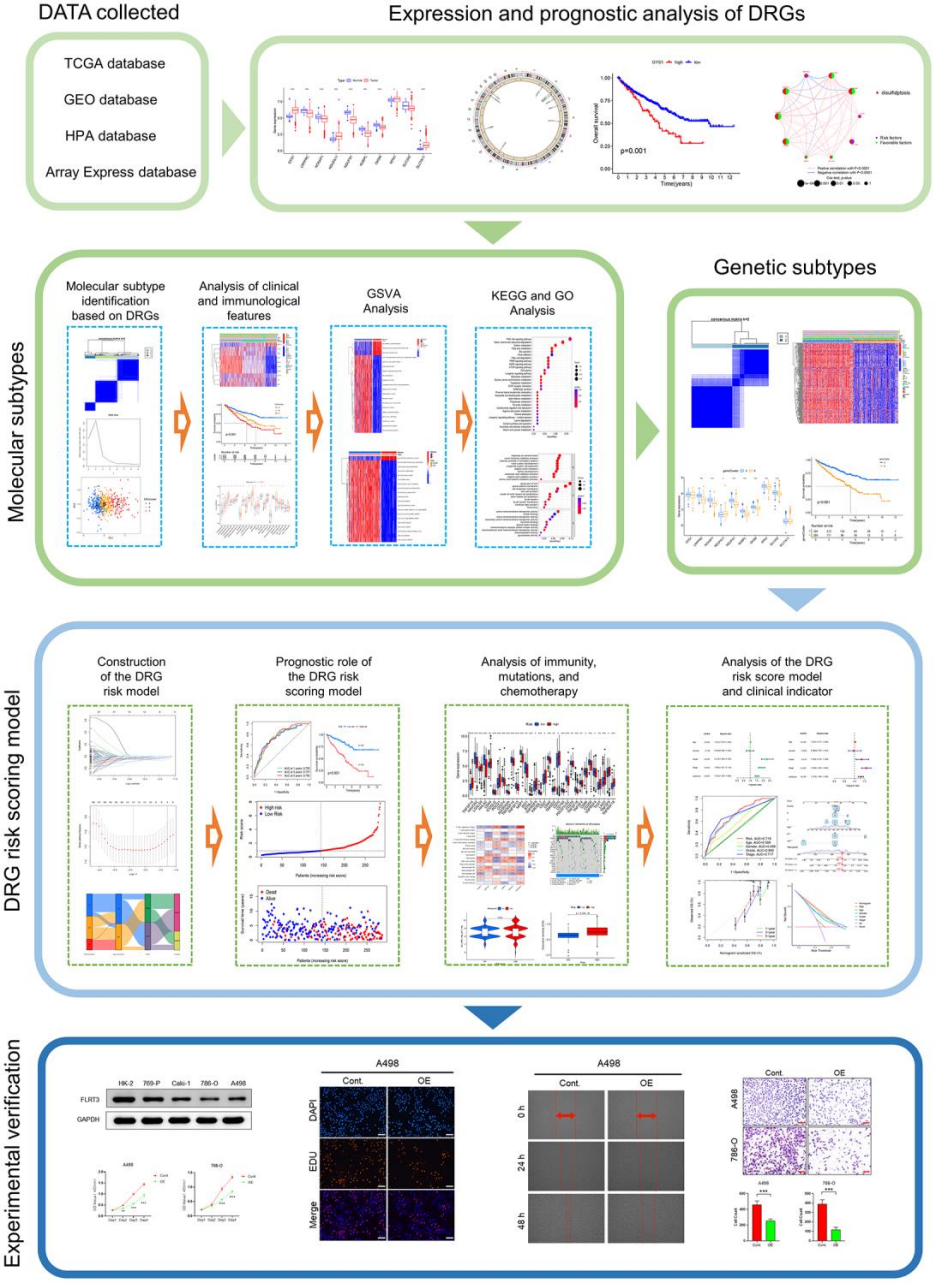
The flowchart of this study.

**Figure 2:**
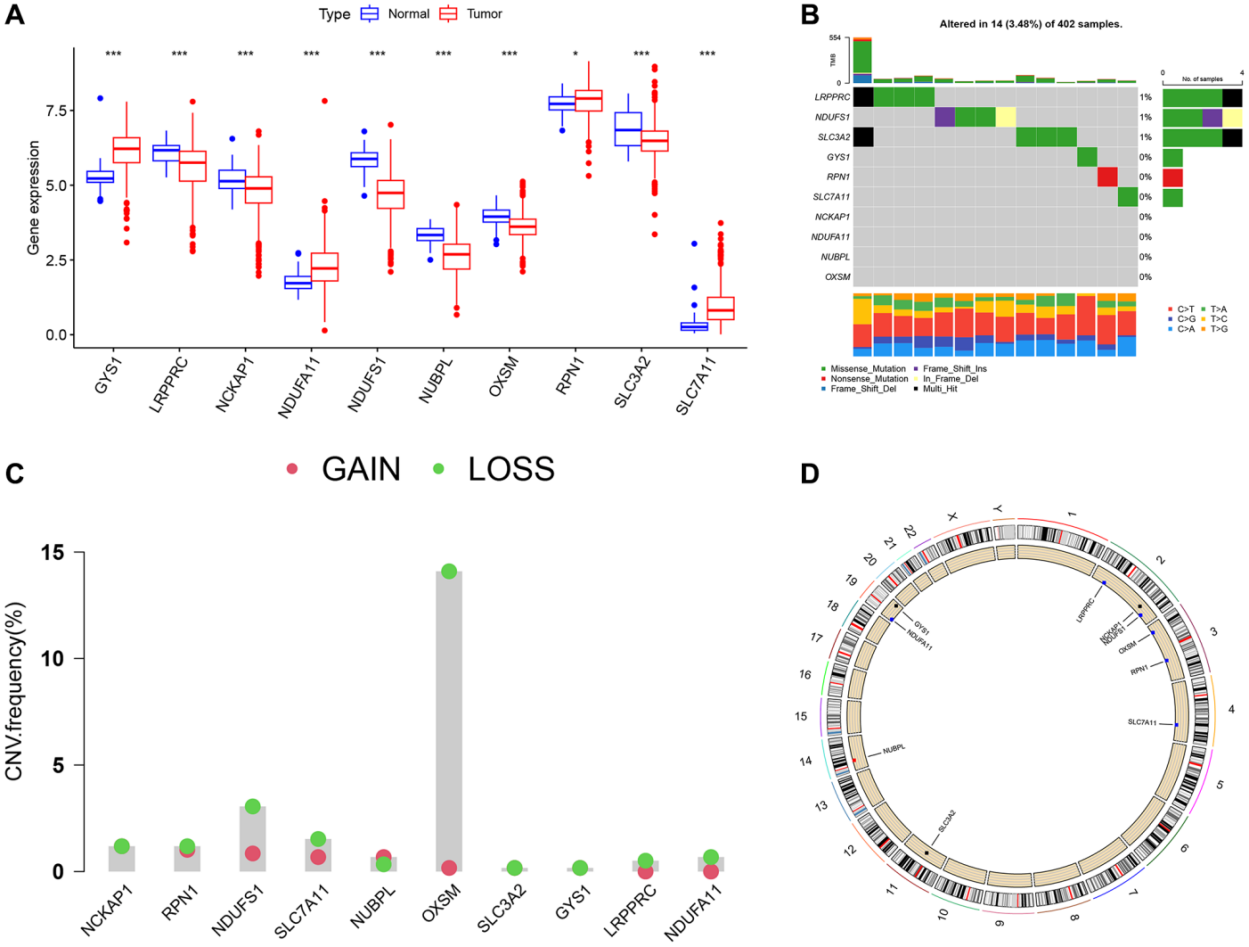
**Differential expression and genetic alteration of DRGs in ccRCC.** (**A**) Expression of DRGs in ccRCC and normal tissues. (**B**) The frequency of somatic mutations in DRGs in ccRCC. (**C**) CNV of DRGs in ccRCC. (**D**) The detailed location of CNV alterations on human chromosomes.

A detailed flowchart of this study is displayed in Figure 1. First, we examined the expression of disulfidptosis-related genes (DRGs) in the TCGA-KIRC dataset and found that all 10 DRGs were differentially expressed in tumor and normal samples. Among them, the expression of GYS1, NDUFA11, RPN1, and SLC7A11 were elevated in ccRCC samples, but the expression of LRPPRC, NCKAP1, NDUFS1, NUBPL, OXSM, and SLC3A2 were decreased in normal samples (Figure 2A). The high expression of SLC7A11 in ccRCC satisfied the prerequisites for the occurrence of disulfidptosis and laterally indicated the availability of disulfidptosis as a specific cell death pattern in ccRCC. Somatic mutation analysis showed (Figure 2B) that only 14 samples (3.48%) were mutated out of 402 ccRCC samples from TCGA. Next, we performed copy number variation (CNV) frequency analysis on DRGs, and OXSM had the highest frequency of CNV loss (Figure 2C). The CNV alterations of DRGs on chromosomes are shown in Figure 2D.

### Relationship between DRGs and prognosis

**Figure 3:**
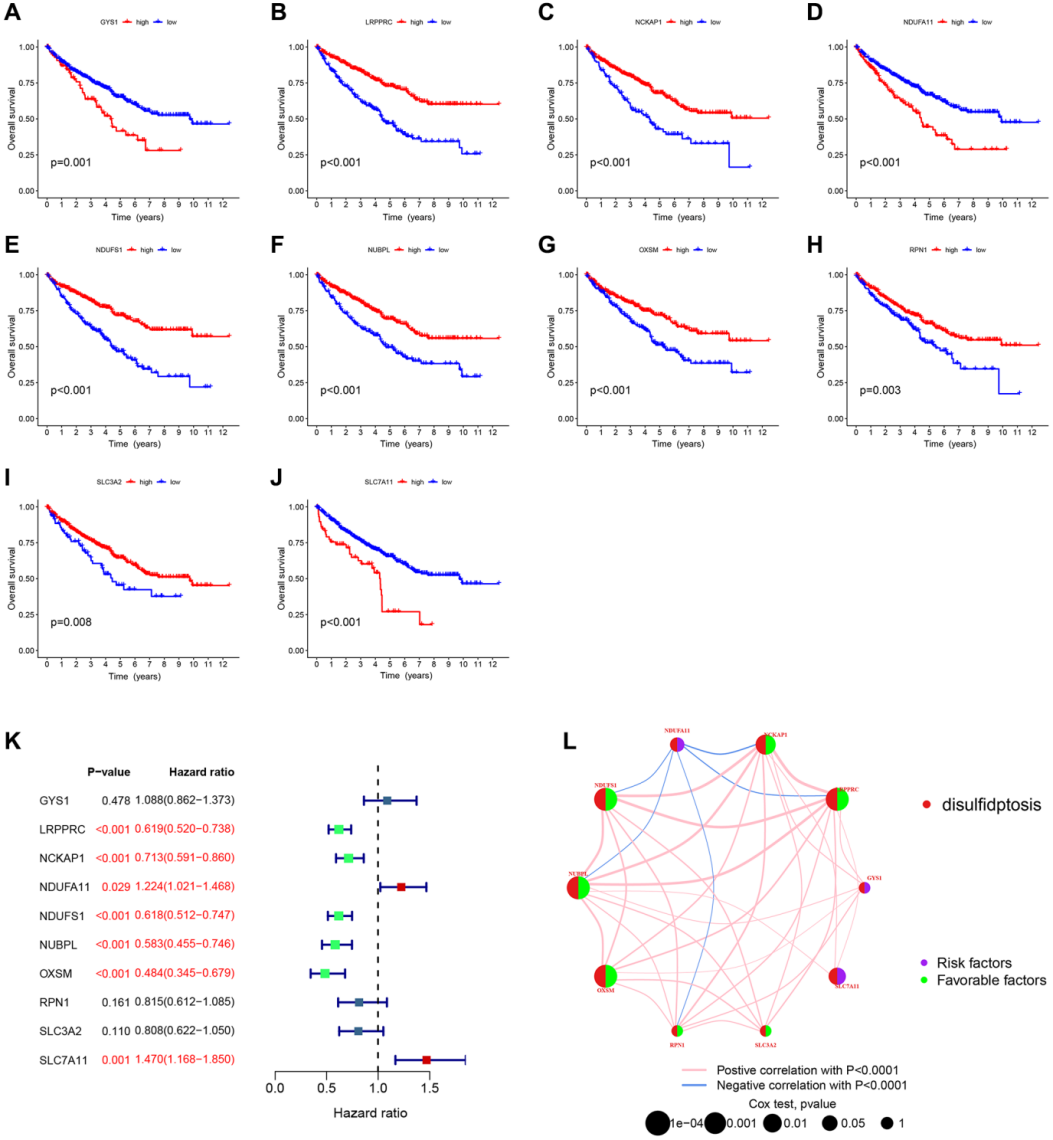
**Relationship between DRGs and prognosis.** (**A**–**J**) Kaplan-Meier plots illustrated the relationship between DRGs and overall survival (OS). (**K**) COX forest plots illustrated the relationship between DRGs and patient prognosis. (**L**) Prognostic network plots for DRGs. The line connecting two DRGs represented their interaction, and the thicker the line, the stronger the association. Positive correlation was depicted by the pink line, while negative correlation was represented by the blue line.

We used KM and Cox analyses to assess the prognostic value of DRGs in the combined TCGA-GSE29609 dataset. The results showed that all 10 DRGs were associated with overall survival (OS) (Figure 3A–3J). The results of the Cox analysis were shown in the forest plot (Figure 3K), and 7 DRGs were associated with prognosis. Figure 3L showed the prognostic network diagram of DRGs, which demonstrated that GYS1, SLC7A11, and NDUFA11 are risk factors for ccRCC patients. LRPPRC, NCKAP1, NDUFS1, NUBPL, OXSM, RPN1, and SLC3A2 are protective factors for ccRCC patients. These results suggest that DRGs were significantly associated with the survival of ccRCC patients. Therefore, we hypothesized that disulfidptosis may be a potential target for ccRCC treatment and performed subsequent analysis.

### Identification of DRG-related molecular subtypes and its GSVA and immunological analysis

**Figure 4:**
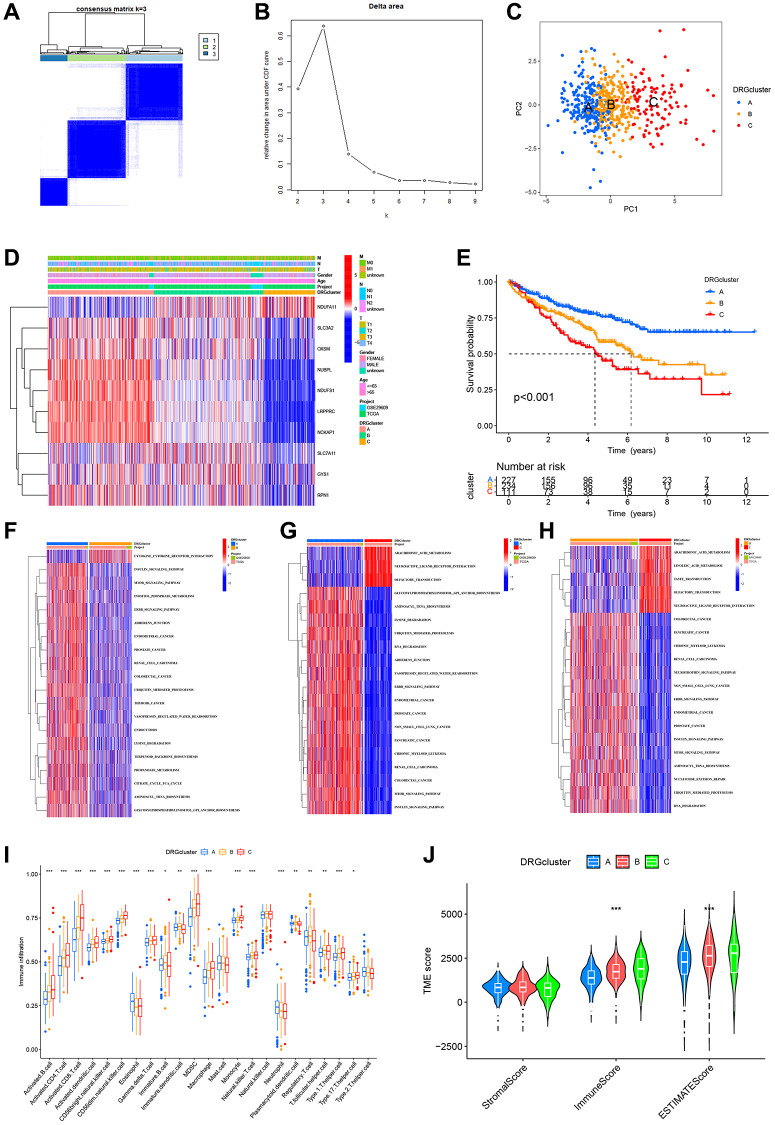
**Identification of DRG-related molecular subtypes and its GSVA and immunological analysis.** (**A**) Consistency matrix for the three clusters. (**B**) The cumulative distribution function based on the sign. (**C**) Principal component analysis. (**D**) Heat map showing the clinical characteristics of molecular subtypes. (**E**) KM survival curves for three molecular subtypes. (**F**–**H**) The GSVA heat map displayed the discrepancy in pathways between different molecular subtypes. (**I**) Differences in immune cell infiltration between different molecular subtypes. (**J**) TME scores for the 3 molecular subtypes.

To clear the expression patterns of the 10 DRGs in ccRCC and their potential biological roles, we typed ccRCC patients using consensus cluster analysis. The consistency matrix of subtypes worked best when K=3, so we classified ccRCC patients into three DRG-related molecular subtypes: DRGcluster A (*n* = 227), DRGcluster B (*n* = 234), and DRGcluster C (*n* = 111) (Figure 4A, 4B). Principal component analysis showed a significantly different distribution among the three clusters (Figure 4C). Figure 4D demonstrated the association of the three molecular subtypes with the expressions of DRGs, patient gender, age, and TNM stage. Previous studies identified SLC7A11, SLC3A2, RPN1, and NCKAP1 as the most relevant genes promoting disulfidptosis [6], and notably, these genes were more expressed in DRGcluster A than in the other two subgroups and least expressed in DRGcluster C. Therefore, we suggested that DRGcluster A is the disulfidptosis activating subgroup and DRGcluster C is the disulfidptosis inhibiting subgroup. The survival analysis showed that patients in DRGcluster A had the best prognosis, followed by those in DRGcluster B and the worst in DRGcluster C (Figure 4E). This result leads us to speculate that the activation of disulfidptosis is beneficial to the prognosis of patients. Next, we performed a GSVA analysis of the molecular subtypes of DRGs and identified significant molecular functional differences between the three clusters. Figure 4F–4H showed the 20 pathways that differed most significantly between the three clusters. DRGcluster C with the worst prognosis had significantly higher ARACHIDONIC ACID METABOLISM than DRGcluster A and B. DRGcluster A with the best prognosis had more active GLYCOSYLPHOSPHATIDYLINOSITOL GPI ANCHOR BIOSYNTHESIS, LYSINE DEGRADATION AMINOACYL TRNA BIOSYNTHESIS, UBIQUITIN MEDIATED PROTEOLYSIS, ERBB SIGNALING PATHWAY than DRGcluster B and C. The GO GSVA analysis results also revealed notable differences among the three clusters (Supplementary Figure 1). These differences in molecular function may account for the different prognoses between molecular subtypes.

We next examined the association between molecular subtypes and tumor immunity. Analysis of ssGSEA revealed that the vast majority of immune infiltrating cells differed among the three molecular subtypes, with the majority of immune cells being more abundant in DRGcluster C, which corresponded to the TME score results (Figure 4I, 4J).

### Identification of DRG-related genetic subtypes and their prognostic value

**Figure 5:**
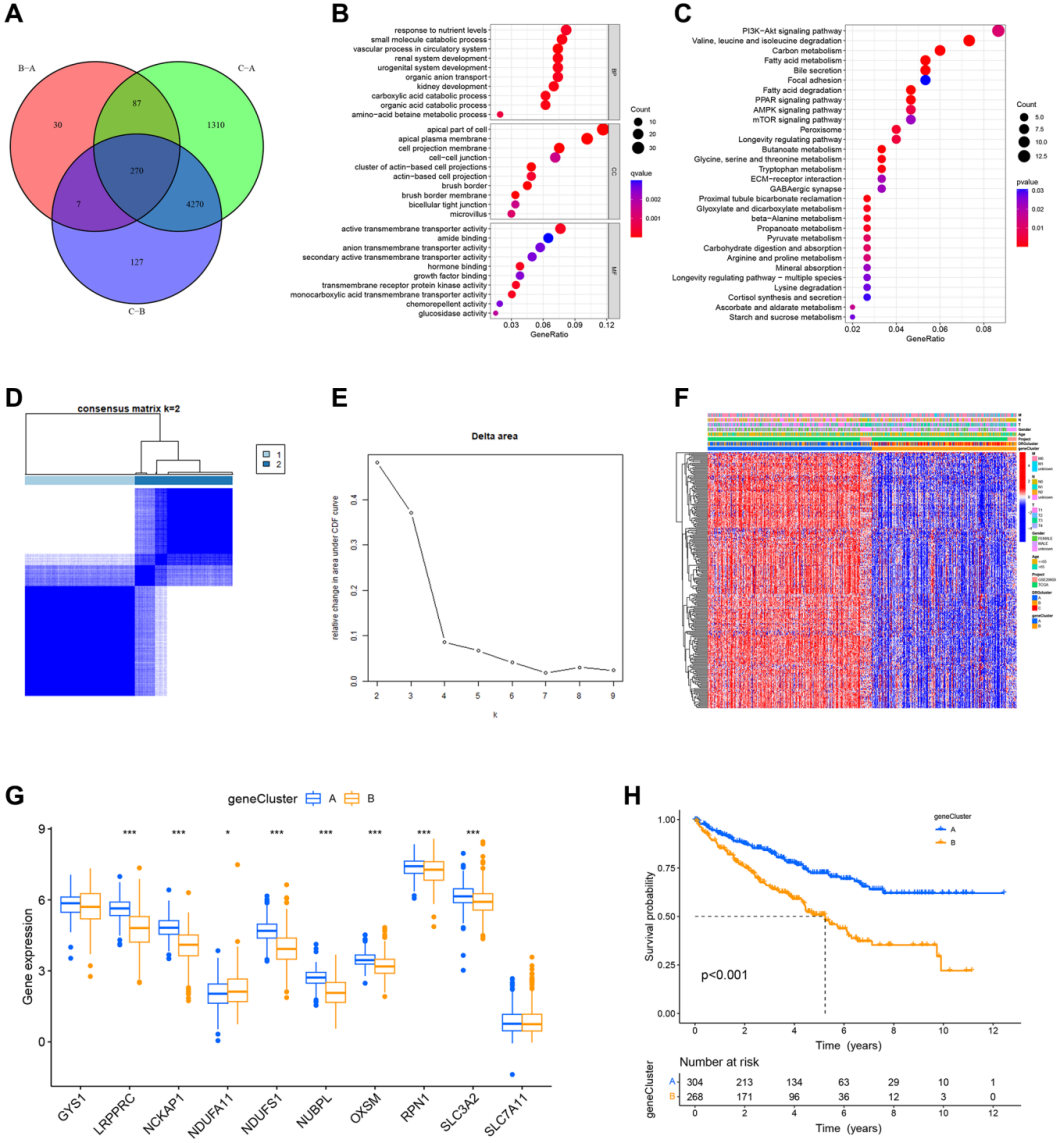
**Identification of DRG-related genetic subtypes and their prognostic value.** (**A**) Venn plot of differentially expressed genes between molecular subtypes. (**B**, **C**) GO and KEGG enrichment analysis of DEGs. (**D**, **E**) Two genetic subtypes (k = 2) were identified by consensus clustering analysis based on the expression of 268 prognosis-related DEGs. (**F**) Heat map illustrating the gene landscape in genetic subtypes A and B and its association with clinical characteristics. (**G**) DRGs are differentially expressed in different genetic subtypes. (**H**) Survival analysis of two genetic subtypes.

To further explore the potential biological behavior of different DRG-related molecular subtypes, we identified 270 differentially expressed genes (DEGs) among these three subtypes (Figure 5A). In addition, we sorted out the important biological functions and pathways of DEGs using GO and KEGG enrichment analysis. According to GO analysis, DEGs were closely associated with biological processes that renal system development, urogenital system development, response to nutrient levels, small molecule catabolic process and active transmembrane transporter activity (Figure 5B). The KEGG results indicated that DEGs are involved in PI3K−Akt signaling pathway, valine, leucine, and isoleucine degradation, and Carbon metabolism et al. (Figure 5C). Considering the outcomes of the aforementioned enrichment analysis, we hypothesize that disulfidptosis is closely associated with the development of ccRCC and its metabolism.

Then, 268 genes associated with prognosis were chosen by univariate Cox regression analysis of DEGs (*p* < 0.05). Based on these prognosis-related DEGs, ccRCC patients were clustered into different DRG-related genetic subtypes by performing a second consensus clustering. Because clustering was best at K = 2, we classified patients into geneCluster A (*n* = 304) and geneCluster B (*n* = 268) (Figure 5D, 5E). Figure 5F displays, in heat map form, the genetic landscape of gene subtypes and their relationship with patients’ TNM stage, gender, age, and molecular subtypes. The geneCluster A and geneCluster B differed significantly in the expression of DRGs (Figure 5G), and survival analysis showed that geneCluster A had better OS than geneCluster B (Figure 5H).

### Construction of the prognostic DRG risk scoring model

**Figure 6:**
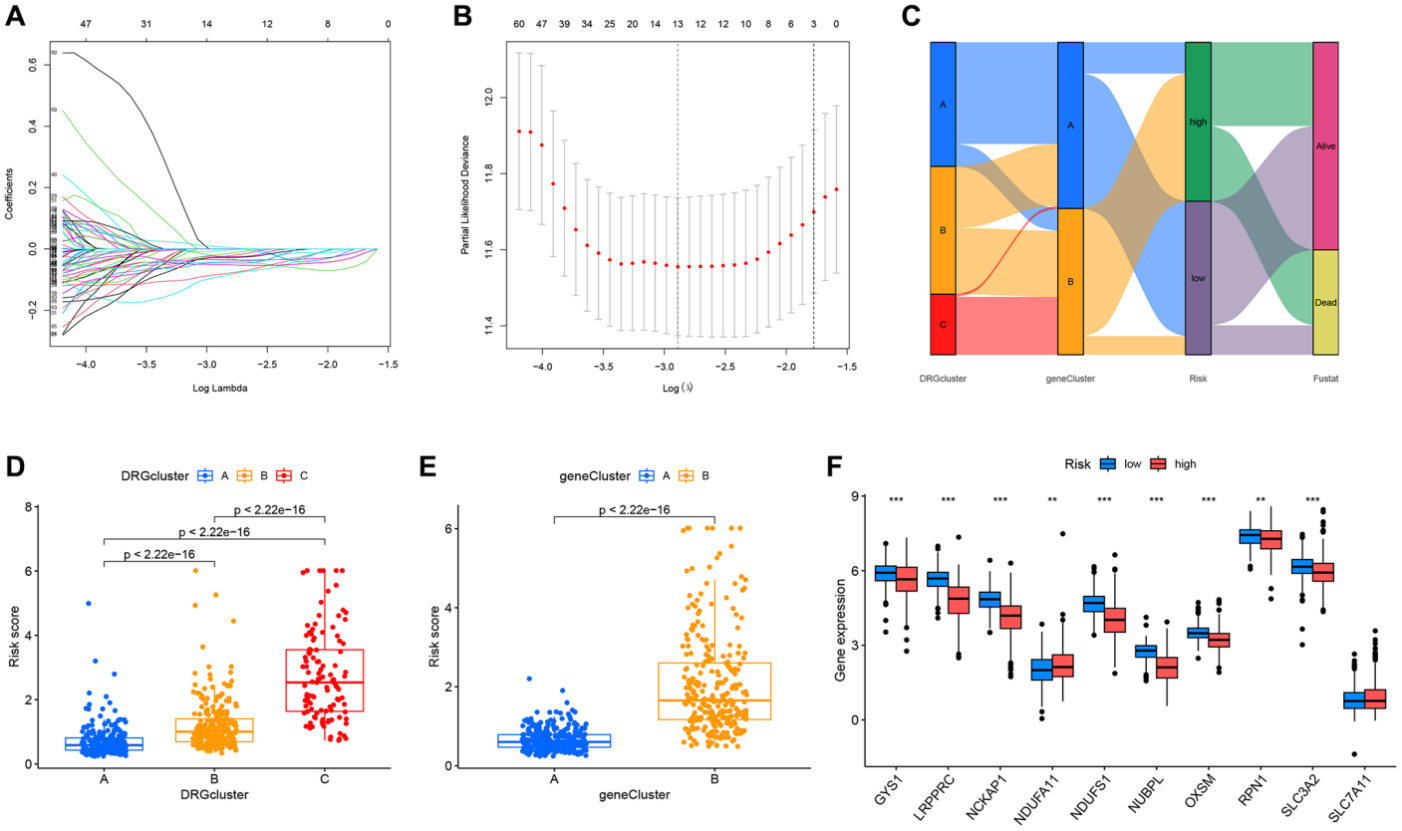
**Construction of the prognostic DRG risk scoring model.** (**A**, **B**) LASSO regression analysis. (**C**) Sankey diagram showing the relationship between different molecular subtypes, genetic subtypes, risk scores, and survival outcomes. (**D**, **E**) Respective risk scores of different molecular subtypes and genetic subtypes. (**F**) Box plot displaying the analysis of differences in DRGs expression between the high and low-risk groups.

Risk models can be used to predict the prognosis of patients and also to select more appropriate treatments for patients based on the expression of risk genes. Therefore, we developed a DRG risk scoring model. First, patients from the combined TCGA-GSE29609 dataset were stratified into the training group (*n* = 286) and the testing group (*n* = 286) randomly. Based on 268 prognosis-related DEGs, the LASSO algorithm was used to find the optimum prognostic gene (Figure 6A, 6B). Then we selected the five most key risk scoring genes (FLRT3, ATP1A1, SAA1, PDK4, and KCNJ15) by multivariate Cox analysis to create the DRG risk scoring model in the training group. Risk score = expFLRT3 × (−0.113081065) + expATP1A1 × (−0.188851917) + expSAA1 × 0.048476033 + expPDK4 × (−0.175069518) + expKCNJ15 × (−0.11452586). Patients with ccRCC were stratified into high-risk and low-risk groups according to their median risk scores. The Sankey diagram illustrates the process by which the patients were stratified into various molecular subtypes, genetic subtypes, risk score groups, and their survival status (Figure 6C). Through calculating the risk scores for each ccRCC patient in different molecular and genetic subtypes, we found that for the molecular subtypes, DRGcluster C had the highest risk score, DRGcluster B followed, and DRGcluster A had the lowest (Figure 6D). Among the genetic subtypes, geneCluster B had a remarkably higher risk score than geneCluster A (Figure 6E). This was consistent with the previous prognostic analysis. Figure 6F displayed the DRG expression of the two risk groups, and there were significant differences in 9 out of 10 DRGs.

### Validating the prognostic role of the DRG risk scoring model

**Figure 7:**
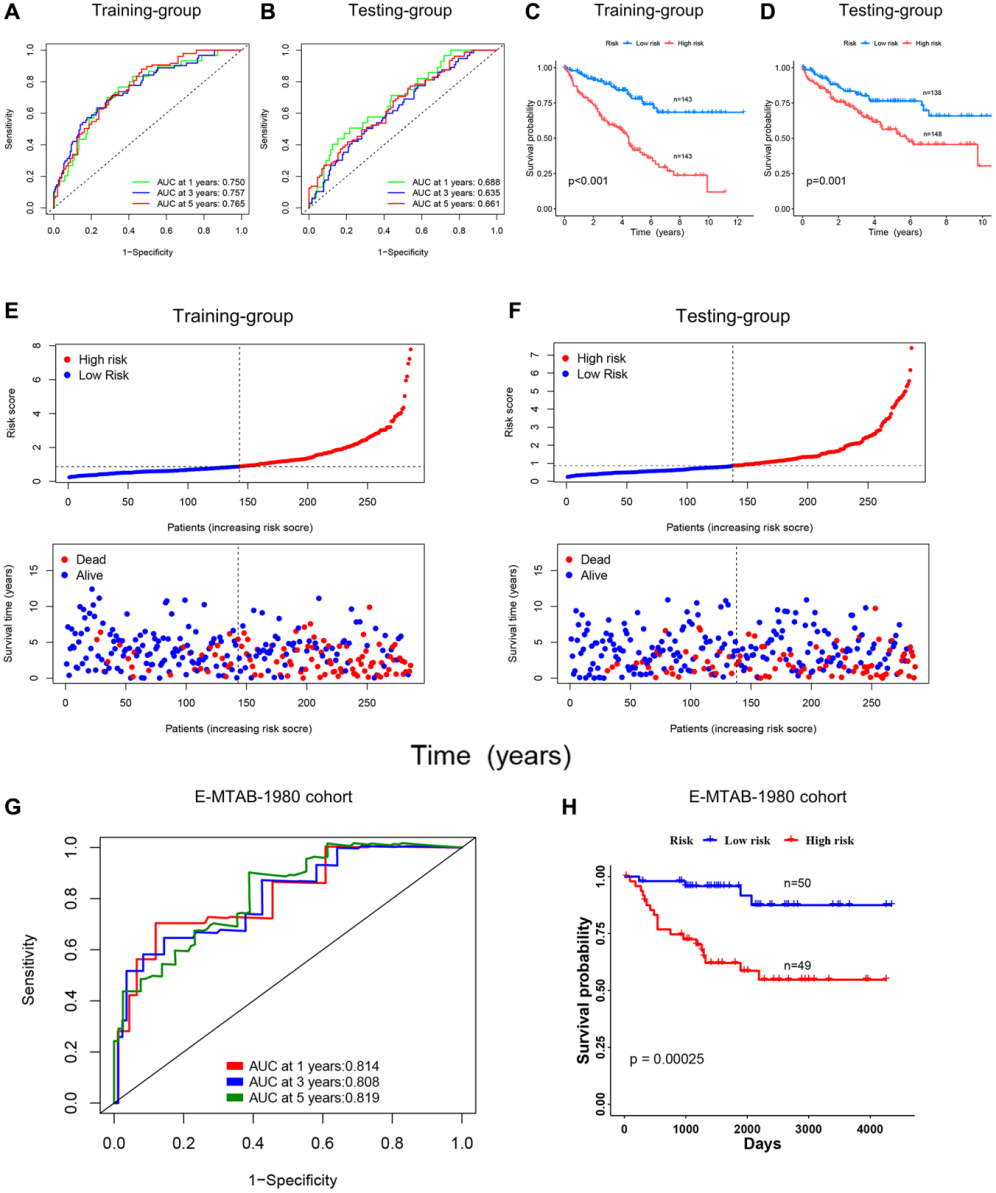
**Validating the prognostic role of the DRG risk scoring model.** (**A**, **B**) ROC curves of training and testing-group for predicting 1, 3, and 5-year survival. (**C**, **D**) KM curves of training and testing-group. (**E**, **F**) Point and scatter plots of risk score distribution and patient survival in training and testing-group. (**G**, **H**) ROC curves and KM curve of E-MTAB-1980 cohort.

We further verified the risk scoring model’s predictive efficacy. Figure 7A, 7B showed the receiver operator curves (ROC) of the risk scoring model to predict survival status at 1, 3, and 5 years, which represent the specificity and sensitivity of the risk scoring model. It can be seen that the area under the curve (AUC) exceeded 0.6 in the training and testing groups. The OS of the high-risk and low-risk groups was compared using the KM curve, and the results of the training and testing groups revealed that the low-risk group’s OS was clearly better (Figure 7C, 7D). For patients in the training and testing groups, survival time decreased with increasing risk scores, as shown in Figure 7E, 7F. The prognostic analysis for the merged group was summarized in Supplementary Figure 2A–2D. The outcomes were consistent with the training and testing groups. In addition, we validated the risk score model using the E-MTAB-1980 cohort, and the results showed that the DRG risk score model also had good predictive performance in this cohort. As shown in Figure 7G, the AUC values of the DRG risk score model at 1, 3, and 5 years in the E-MTAB-1980 cohort were 0.814, 0.808, and 0.819, respectively. Moreover, patients with high-risk scores in the cohort showed a worse prognosis (Figure 7H). We also compared the 1-, 3-, and 5-year AUC values of the DRG risk scoring model with the AUC values of 4 other published prognostic models (for ccRCC patients in the TCGA database) to test the prognostic performance of our model [13–16]. The results showed that our prognostic model exhibited better performance (Supplementary Figure 2E). The results above showed that the DRG risk scoring model can reliably forecast the prognosis of patients with ccRCC.

### Relationship between the DRG risk scoring model and immune cell, immune checkpoints, somatic mutations, TMB, and immunotherapy

**Figure 8:**
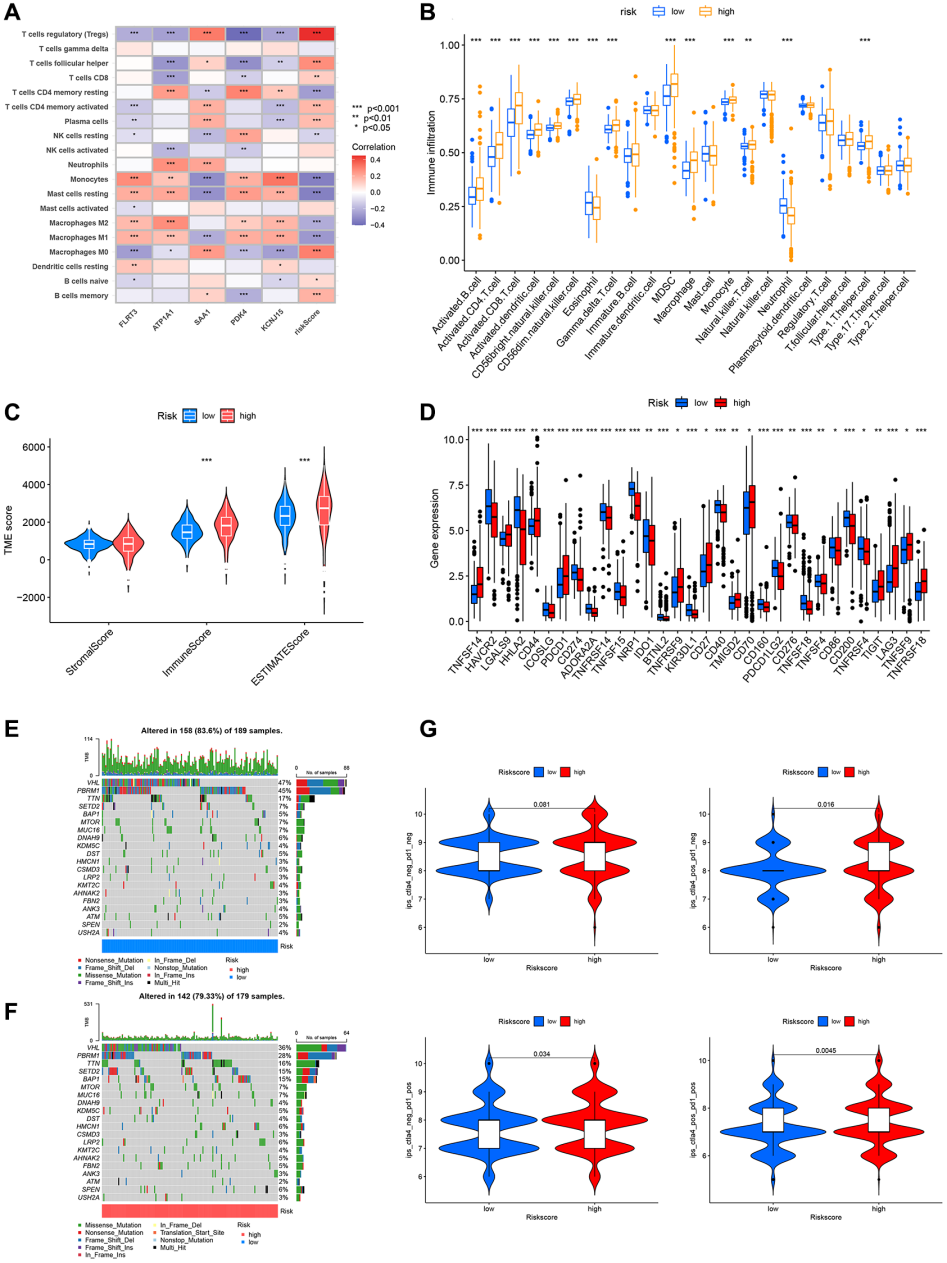
**Relationship between DRG risk scoring model and immune cell, immune checkpoints, somatic mutations, TMB, and immunotherapy.** (**A**) The association between risk scoring genes, risk score, and immune cells. (**B**) Differences in immune cell infiltration between high and low-risk groups. (**C**) TME scores for high and low-risk groups. (**D**) The expression of immune checkpoint genes in the high-risk and low-risk groups. (**E**, **F**) Frequency of somatic mutations in high and low-risk groups. (**G**) IPS results from the TCIA database for high and low-risk groups. *P* < 0.05 was considered statistically significant.

In recent years, tumor immunotherapy has advanced significantly [17]. Immune cell infiltration, immune checkpoints, somatic mutations, and tumor mutation burden (TMB) can influence the efficacy of immunotherapy [18–21]. Hence, we analyzed them one by one. From Figure 8A, we can understand that the association was very tight between risk scoring genes, risk score, and immune cells. Additionally, there was substantial diversity in immune cell infiltration between the high and low-risk groups. For instance, the high-risk group had substantially more activated CD4 T cells, activated CD8 T cells, and natural killer T (NKT) cells infiltrate than the low-risk group (Figure 8B). Patients in the high-risk group had higher percentages of activated CD4 T cells and CD8 T cells (Supplementary Figure 3). TME scoring indicated that the immune and total scores in the high-risk group were considerably higher (Figure 8C). Moreover, we examined the expression of checkpoint genes in the two risk groups. A total of 32 immune checkpoint genes showed statistically significant differential expression in the two risk groups (Figure 8D). The distribution of somatic mutations between the high and low-risk scoring groups in the TCGA-KIRC set was shown in detail by waterfall plots (Figure 8E, 8F). The frequency of VHL and PBRM1 mutations was significantly lower in the low-risk group. TMB is closely related to immunotherapy of tumors, and patients with higher TMB may have better results with immune checkpoint inhibitors (ICIs) [22]. As shown in the figure, although the TMB of patients in the high-risk group was higher than that of the low-risk group, the results were not statistically significant and more data are needed for validation (Supplementary Figure 4). Next, we forecasted the response to immunotherapy by the IPS according to the TCIA database. The results revealed that the high-risk group had more patients who were PD-1 single positive, CTLA-4 single positive, or PD-1/CTLA-4 double positive, suggesting that these patients may benefit more from immunotherapy (Figure 8G).

### Drug susceptibility analysis

**Figure 9:**
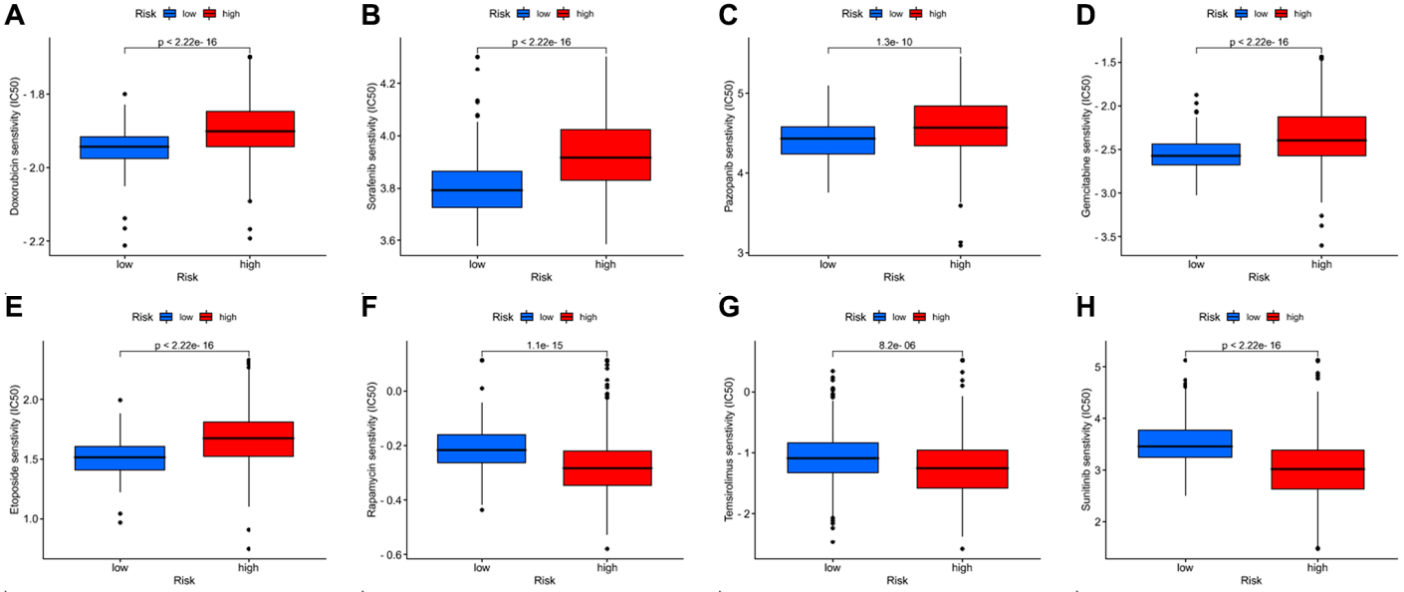
**Drug susceptibility analysis for chemotherapeutic agents commonly used in the clinic.** (**A**–**H**) Doxorubicin, sorafenib, pazopanib, gemcitabine, etoposide, rapamycin, temsirolimus, and sunitinib.

We examined the efficacy of different chemotherapeutic drugs for patients in the two risk groups. The IC50 was calculated by the “pRRophetic” package, and the results include not only the chemotherapeutic drugs we used clinically but also those in clinical trials. We list all of the chemotherapeutic drugs for which there were statistically significant differences in chemosensitivity between the two risk groups (Supplementary Table 1). For chemotherapeutic drugs commonly used in the clinic [1, 23, 24], rapamycin, temsirolimus, and sunitinib may have higher sensitivity in ccRCC patients with a high DRG risk score. Patients with a low DRG risk score, on the other hand, were more sensitive to doxorubicin, sorafenib, pazopanib, gemcitabine, and etoposide (Figure 9A–9H). For a particular ccRCC patient, we can predict which drugs are more effective for the patient’s treatment and make a choice according to the DRG risk score.

### Analysis of the DRG risk score model and clinical indicators

**Figure 10:**
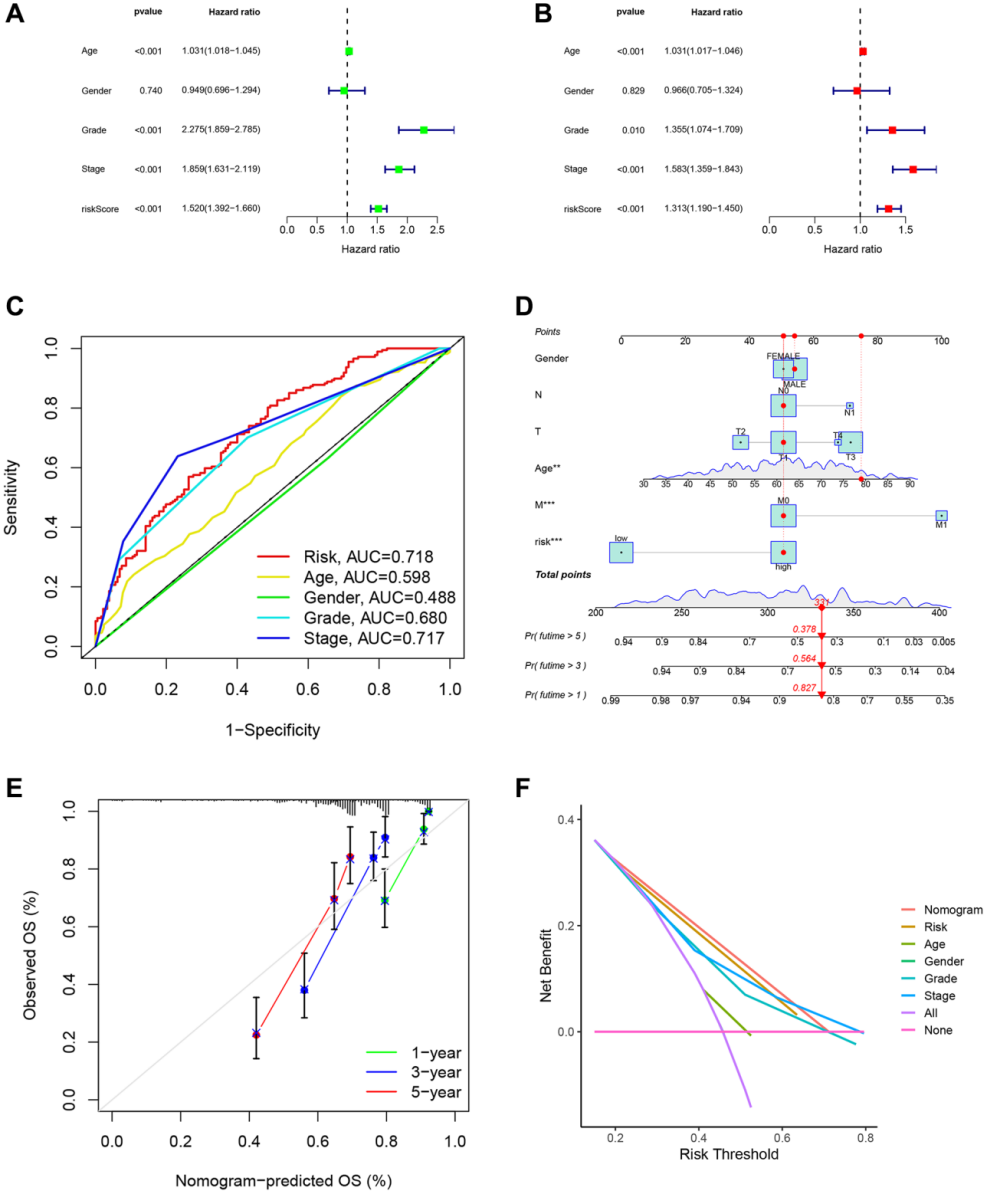
**Analysis of the DRG risk score model and clinical indicator.** (**A**) Univariate regression analyses demonstrated the risk model as an independent prognostic indicator. (**B**) Multivariate regression analyses demonstrated the risk model as an independent prognostic indicator. (**C**) The clinROC curves with risk score models and common clinical indicators. (**D**) The nomogram predicted patient OS in the 1st, 3rd, and 5th years. (**E**) The calibration curves displayed the accuracy of the nomogram in the 1st, 3rd, and 5th years. (**F**) The DCA analysis of different indicators.

We performed univariate and multivariate Cox regression analysis of the risk model in the TCGA-KIRC cohort and confirmed that the prognostic model was an independent predictor (Figure 10A, 10B). In addition, the clinROC curve of the model had an AUC value of 0.718, indicating its superiority to commonly used clinical indicators (Figure 10C). We have proven the accuracy and importance of the DRG risk scoring model in forecasting the prognosis of ccRCC patients, and we further created a nomogram containing DRG risk score, age, and TNM staging to predict ccRCC patients’ OS at 1, 3, and 5 years (Figure 10D). We tested the accuracy of the nomogram by calibrating the graph, and our constructed nomogram had good accuracy in ccRCC patients compared with the ideal model (Figure 10E). Finally, we performed a DCA analysis, which showed that the nomogram and risk models outperformed the commonly used clinical indicators (Figure 10F).

### Risk scoring genes were abnormally expressed in ccRCC

**Figure 11:**
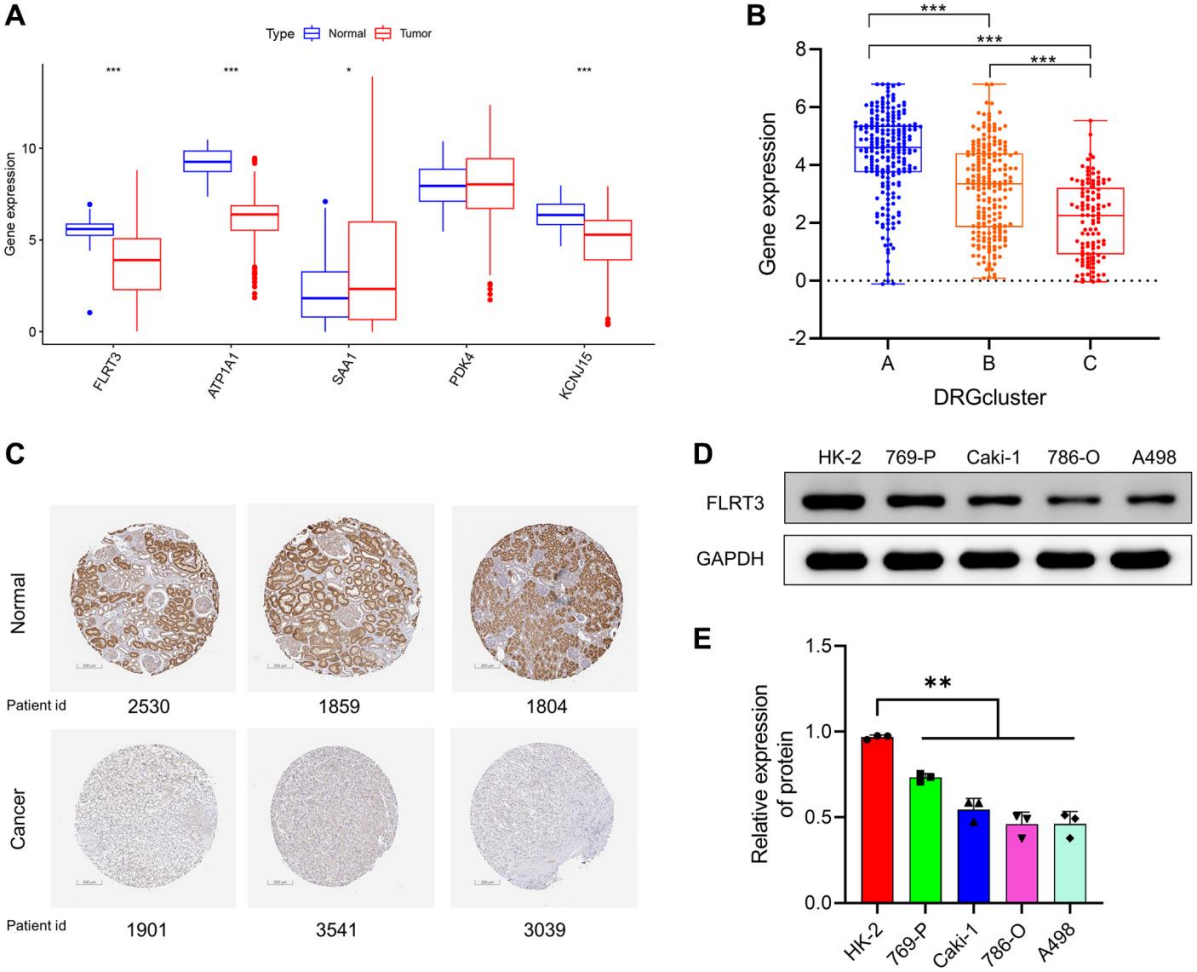
**Risk scoring genes were abnormally expressed in ccRCC.** (**A**) Expression levels of risk scoring genes between tumor and normal samples according to TCGA. (**B**) FLRT3 is differentially expressed in different DRG-related molecular subtypes. (**C**) Representative IHC images of FLRT3 from the Human Protein Atlas (HPA) database for normal and tumor samples. (**D**, **E**) Analysis of FLRT3 expression in ccRCC cells and normal kidney cells by Western blotting assay.

We further explored the role of risk scoring genes. First, we found that four of the five risk scoring genes were abnormally expressed in ccRCC according to the TCGA database. Among them, FLRT3, ATP1A1, and KCNJ15 were expressed at decreased levels in tumor samples, and the expression of SAA1 was increased in tumor samples (Figure 11A). Overall survival analysis of risk scoring genes revealed that ccRCC patients with high expression of FLRT3, ATP1A1, PDK4, and KCNJ15 presented better prognosis, but patients with high SAA expression presented worse prognosis (Supplementary Figure 5), which was consistent with the positive and negative risk coefficients in the risk scoring model.

FLRT3 is abnormally expressed in ccRCC and negatively correlated with poor prognosis of patients. Among the DRG-related molecular subtypes, FLRT3 expression was highest in the disulfidptosis activation subgroup (DRGcluster A) and lowest in the disulfide death inhibition subgroup (DRGcluster C) (Figure 11B). It indicates that FLRT3 expression is associated with the promotion of disulfidptosis. Because there were no studies have discussed the role of FLRT3 in ccRCC and given the importance of FLRT3, we decided to investigate FLRT3 at a deeper level to eliminate this knowledge blindness. Using the Human Protein Atlas (HPA) database, we further validated that FLRT3 expression was reduced in ccRCC. Figure 11C showed a typical immunohistochemical staining image from the HPA database. Then, we used Western blotting experiments to verify that ccRCC cells expressed FLRT3 less than normal kidney cells (Figure 11D, 11E).

### FLRT3 overexpression inhibits proliferation and metastasis of ccRCC cells

**Figure 12:**
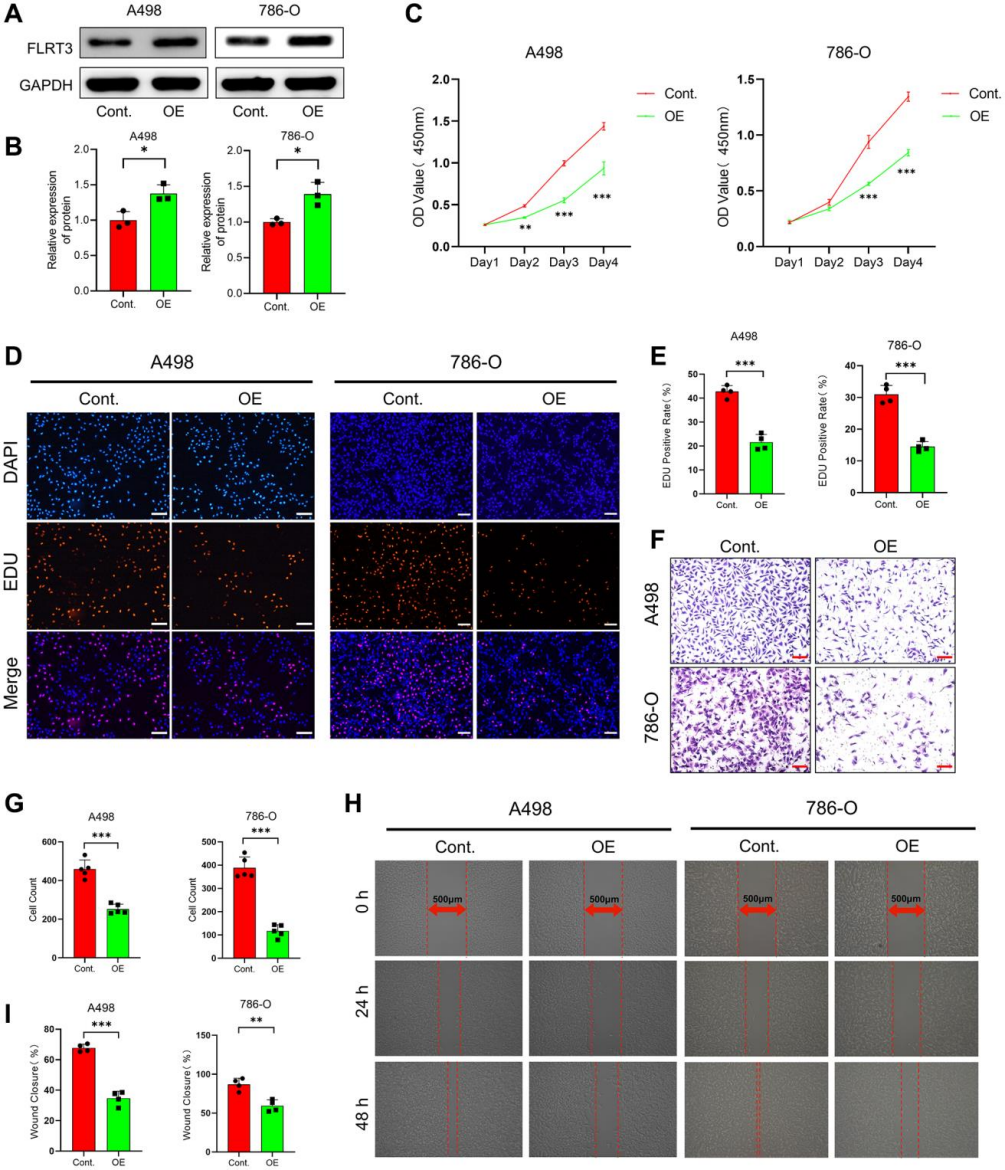
**FLRT3 overexpression inhibits the proliferation and metastasis of ccRCC cells.** (**A**, **B**) The Western blot experiment demonstrated that FLRT3 was overexpressed in the OE group. (**C**) CCK8 assay showed that FLRT3 overexpression led to reduced proliferation capacity of A498 and 786-O cells. (**D**, **E**) The results from EdU incorporation experiments showed that FLRT3 overexpression resulted in a significant reduction in the proliferation capacity of A498 and 786-O cells. (**F**, **G**) The transwell assay illustrates that FLRT3 overexpression significantly impairs the invasive ability of A498 and 786-O cells. (**H**, **I**) Wound healing assays illustrated that elevated FLRT3 expression caused a decrease in the migratory capacity of A498 and 786-O cells. Scale bar: 500 μm.

We examined the impact of FLRT3 overexpression on the biological function of ccRCC cells by plasmid transfection. The FLRT3 protein was significantly elevated in ccRCC cells after transfection (Figure 12A, 12B). Through CCK8 assay and EdU incorporation assay, we found that FLRT3 overexpressing ccRCC cells had a significantly reduced proliferative capacity (Figure 12C–12E). The influence of overexpression of FLRT3 on the migration and invasion in ccRCC cells was evaluated too. As shown in Figure 12F–12I, the invasive and migratory abilities of ccRCC cells in the OE group were considerably reduced. The results suggest that overexpression of FLRT3 inhibits the proliferation and metastasis of ccRCC cells. This result could be caused by promoting disulfidptosis, but more experiments are needed to verify this.

## DISCUSSION

The morbidity of renal cell carcinoma (RCC) is increasing worldwide, and the mortality rate is about 20% [25]. Currently, RCC is broadly classified into 3 main histological subtypes: clear cell RCC (ccRCC), papillary RCC, and chromophobe RCC [26, 27]. Within them, ccRCC is the most usual (70–80% of all types) and the most lethal type of renal cell carcinoma [28]. With advances in technology, many new diagnostic techniques and treatment modalities have enhanced the early ccRCC patients’ prognosis, but the overall survival of ccRCC is still depressing [3, 29]. Hence, it is necessary to find novel biomarkers for ccRCC prognosis prediction and therapeutic targeting. Disulfidptosis is a newly characterized mode of cell death, and it differs from the known ways of cell death such as apoptosis [30], ferroptosis [31], or cuproptosis [32]. In simple terms, disulfidptosis refers to the abnormal accumulation of disulfide in SLC7A11 high expressing cells under glucose starvation conditions, which eventually leads to cell death [6]. However, there are not many studies on disulfidptosis and DRGs in various tumors and even fewer in ccRCC. Therefore, we performed a multi-omics study to examine the correlation between DRGs and prognosis and treatment in ccRCC patients.

In this study, we obtained transcriptomic data and matching clinical data of ccRCC patients from the TCGA and GEO databases. By examining the expression of DGRs, we revealed that all 10 DRGs were abnormally expressed in tumor tissues. The results of univariate Cox analysis and KM analysis to analyze patient prognosis showed that the 10 DRGs were also closely associated with ccRCC patients’ prognosis. Hence, we supposed that disulfidptosis could be a possible target for ccRCC treatment and that DRGs may help predict the treatment response and prognosis of ccRCC patients, so we conducted a deeper study. Cancers with similar morphology usually have very different clinical features and different responses to treatment [33], so we classified cancer patients into three molecular subtypes on the basis of the expression of DRGs. Molecular subtype DRGcluster C had the worst overall survival, and GSVA pathway analysis revealed a significant enrichment of arachidonic acid metabolism in DRGcluster C compared to DRGclusters A and B. This is also consistent with many studies reporting that arachidonic acid metabolism promotes tumor progression [34, 35]. At present, immunotherapy plays an increasingly important role in ccRCC, but its therapeutic efficacy depends on the tumor immune microenvironment, so we evaluated immune cell infiltration and TME scoring in different molecular subtypes [36–38]. The outcomes demonstrated that the most of immune cells in DRGcluster C were more enriched and had higher TME scores than the other two types. Next, we further divided ccRCC patients into genetic subtypes A and B based on differentially expressed genes between molecular subtypes. Genetic subtypes also present significantly different gene expression, prognosis, and immune landscapes.

In addition, based on differentially expressed genes among DRG-related molecular subtypes, we generated five risk scoring genes. Among these five risk genes, FLRT3, ATP1A1, PDK4, and KCNJ15 were protective factors and SAA1 was a risk factor. Many previous studies have demonstrated that these genes play an important role in tumors. KCNJ15 downregulation promotes the progression of renal cancer by a process associated with epithelial-mesenchymal transition and metallothionein expression [39]. ATP1A1 is significantly reduced in patients with renal cell carcinoma (RCC), and patients with RCC with positive ATP1A1 expression have a better prognosis [40]. SAA1 can be used as a biomarker to predict advanced renal cancer, and its knockdown can impair the ability of renal cancer cells to proliferate and metastasize [41]. Downregulation of FLRT3 expression promotes an aggressive phenotype in colorectal cancer cells, and downregulation of PDK4 is associated with poor prognosis in hepatocellular carcinoma [42, 43]. Based on these risk genes, we constructed a DRG risk scoring model. The good predictive capability of the model was also verified in the testing group, merged group, and E-MTAB-1980 cohort. Because of the DRG risk scoring model’s good capability in predicting the prognosis of ccRCC patients, we further created a nomogram containing DRG risk score, age, and TNM stage to forecast the OS of ccRCC patients at 1, 3, and 5 years. The establishment of the nomogram helps to stratify ccRCC patients’ prognosis and further promotes the application of the DRG risk scoring model clinically.

For immunity, immune cells and the expression of risk genes or risk scores were significantly correlated. Patients in the high-risk group had higher TME scores. Much research has shown that patients’ responses to immunotherapy are dependent on the level of tumor immune cell infiltration, for example, CD4^+^ T cells, CD8^+^ T cells, and natural killer T (NKT) cells contribute to the immunotherapeutic efficacy of tumors [44–48], whereas myeloid-derived suppressor cells (MDSCs) inhibit the antitumor effects of these immune cells and promote tumor progression [49]. This may explain why high-risk patients have a worse prognosis but might gain more from immunotherapy. The results of drug susceptibility analysis revealed that there were also remarkable diversities in the response to various chemotherapeutic drugs between the two risk groups, which may guide us in clinical work to select chemotherapeutic drugs according to the patient’s conditions.

Notably, we also performed Western blot experiments to validate the reduced expression of FLRT3 (one of the risk scoring genes) in ccRCC cells. Furthermore, we discovered that overexpression of FLRT3 could significantly inhibit the proliferation and metastatic ability of ccRCC cells by *in vitro* experiments. This may have been achieved by disulfidptosis. However, our study had some limitations. We only performed bioinformatics analysis of DRGs in the TCGA-ccRCC, GEO (GSE29609), and Array Express (E-MTAB-1980 cohort) databases, and only partially performed *in vitro* experiments to validate the analysis results, so comprehensive validations of external datasets and more complete experimental verification are needed.

## CONCLUSION

In conclusion, we analyzed the genes associated with disulfidptosis and constructed a novel DRG risk scoring model accordingly. This model can provide novel ideas for doctors to predict patient survival and determine clinical treatment plans. The study also fills a knowledge gap about the role of DRGs in ccRCC.

## MATERIALS AND METHODS

### Data collection

We downloaded expression data for renal clear cell carcinoma (ccRCC) from The Cancer Genome Atlas (TCGA) Database (https://portal.gdc.cancer.gov/). We obtained 614 samples (542 tumor samples and 72 normal samples) by searching KIRC in the TCGA database and processed them for transcripts per million (TPM), normalization, and log2 transformation. Copy number variation (CNV), somatic mutations, tumor mutational burden (TMB), and matching clinical characteristics data for ccRCC samples were also acquired from the TCGA database. To eliminate possible heterogeneity in a single database, we downloaded the series matrix data of GSE29609 from the Gene Expression Omnibus (GEO) database (https://www.ncbi.nlm.nih.gov/geo/) for a total of 39 ccRCC samples and fused it with the TCGA dataset as a combined set. Utilizing the “ComBat” function of the sva package, and removing negative values representing outliers, and removing batch effects from different data sets [50]. When we analyzed gene expression, genes with expression values of 0 were removed, and when survival analyses were performed, patients lacking survival parameters were excluded from further analysis. Clinical details of a total of 576 ccRCC patients were displayed in Supplementary Table 2. Furthermore, we collected expression and clinical data of the E-MTAB-1980 cohort from the Array Express database (https://www.ebi.ac.uk/arrayexpress) to validate the stability of the DRG risk scoring model. E-MTAB-1980 cohort includes 101 RCC samples with accurate clinical survival information.

### Identification of molecular subtypes and genetic subtypes by DRGs

We collected 10 disulfidptosis-related genes (DRGs) from previous publications and summarized them (Supplementary Table 3). Based on these 10 genes and using the R software “ConsensusClusterPlus”, we performed consensus unsupervised clustering analysis to group patients (*n* = 572) into several DRG-related molecular subtypes [51]. Differentially expressed genes (DEGs) among various molecular subtypes were used to classify ccRCC patients into various genetic subtypes by a consensus unsupervised clustering algorithm. The criteria for binning are as follows: first, the number of samples in each group is evenly distributed. Second, the horizontal coordinate of the highest point of the delta area curve. Third, the greater the inter-subgroup variation, the smaller the intra-subgroup variation.

### Functional and pathway enrichment analysis

GSVA analyzes differences in function and pathway between different types [52]. The R packages “enrichplot” and “ggplot2” were used to analyze the gene ontology (GO) and pathway (KEGG) enrichment of DEGs [53].

### Construction of the DRG risk scoring model

All ccRCC patients from the combined set were randomly classified into a training group (*n* = 286) and a testing group (*n* = 286) in a 1:1 ratio by the “caret” package in R. In the training group, we created a DRG risk scoring model that was then verified in the testing, merged groups, and E-MTAB-1980 cohort. First, to lower the danger of overfitting, we used the “glmnet” R package to perform logistic least absolute shrinkage and selection operator (LASSO) Cox regression analysis [54]. Then, multivariate Cox analysis was used to pinpoint five DRG-related risk scoring genes. According to these genes, we established a prognostic DRG risk scoring model. Here are how the detailed DRG risk scores are calculated:


$$DRG\;Risk\;score=\sum_{i=1}^{n}\left(Expi\times Coefi\right)$$

Expi is the expression of the DRG-related risk genes. Coefi indicates its risk factor. The details were displayed in Supplementary Table 4. On the basis of their median risk scores, patients were divided into high-risk and low-risk categories. Survival analysis was then carried out, and receiver operating characteristic (ROC) curves were created. Correlations between each subtype, prognosis, and risk score were analyzed using the “GGalluvial” R package [55]. Finally, we constructed a nomogram scoring system including risk score, age, and tumor TMN stage. Moreover, calibration curves at years 1, 3, and 5 were drawn to confirm the nomogram’s correctness. The DCA analysis was performed using the R package “ggDCA” to demonstrate the performance of the model and nomogram by comparing them with common clinical indicators.

### Analysis of prognosis and immune landscape

The prognostic value of different subgroups was assessed by Kaplan-Meier (KM) analysis and the univariate/multivariate Cox regression algorithm by R package “survival”. The R package “ESTIMATE” can calculate TME scores by gene expression profiles, including stromal, immune, and estimated scores [56]. The TME scores of ccRCC patients were evaluated between respective subtypes. The single sample gene set enrichment analysis (ssGSEA) method was used to quantify the level of immunological infiltration of immune cells in the TME of patients with ccRCC. We used The Cancer Immunome Atlas (TCIA) database to obtain immunophenotypic scoring (IPS) of ccRCC patients and predicted differences in response to immunotherapy by IPS for patients in high and low-risk groups.

### Drug susceptibility analysis

We employed the R package “pRRophetic” to evaluate the 50% maximal inhibitory concentration (IC50) by ridge regression for the two risk groups to predict differences in chemotherapeutic susceptibility [57].

### Immunohistochemistry images of renal and cancer

Immunohistochemistry images were acquired from The Human Protein Atlas (https://www.proteinatlas.org/). The knowledge resource’s whole dataset is open access, enabling researchers studying the human proteome from both academia and business to freely access it.

### Cell lines and cell culture

The cell lines used in this research were obtained from the Shanghai Institute of Cell Science, Chinese Academy of Sciences. Human renal normal cells (HK-2) and cancer cell lines (Caki-1, 769-P, A498, and 786-O) were cultured in Dulbecco’s modified Eagle’s medium or RPMI 1640 medium.

### Construction of FLRT3 overexpression cell

The GV230-FLRT3 plasmid designed by Genechem (China) was transfected into A498 and 786-O cells to construct FLRT3-overexpressing cells. Control group cells were transfected with the GV230-vector plasmid. Transfection efficiency was confirmed by Western blot experiments. We referred to the cells overexpressing FLRT3 as the OE group in this research.

### Western blot

Total proteins from renal normal and tumor cells were extracted by total protein extraction reagent, and the different proteins were divided through SDS gel electrophoresis. Then separated proteins were transferred to nitrocellulose membranes. The membranes were blocked at room temperature using 5% skim milk powder for 1 h and then incubated with distinct primary antibodies at 4°C overnight. The membranes were then incubated with the matching secondary antibodies for 1 h at room temperature. Western blot signals and images were obtained by Bio-Rad ChemiDoc XRS chemiluminescence system. The antibodies used were as follows: anti-FLRT3 Ab (#ab223047, 1:2000, Abcam, UK) and anti-GAPDH Ab (#60004-1-Ig, 1:5000, Proteintech, China).

### Cell proliferation ability assay

Cell counting Kit-8 (CCK-8) assay: We examined the changes in cell proliferation ability before and after overexpression of FLRT3 in A498 and 786-O cells. Normal and transfected kidney cancer cells were added to 96-well plates at 1 × 10^3^ cells per well, with 6 replicate wells per group. CCK8 reagent (#K1018, APExBIO Technology, USA) was added 1, 2, 3, and 4 days after cell plating, and 2 h later, the absorbance was measured at 450 nm.

Ethynyl-2-Deoxyuridine (EdU) incorporation assay: 4 × 10^5^ renal cancer cells were seeded into confocal glass dishes per dish, stained according to the instructions of the EdU kit (#KTA2031, Abbkine, USA) when the cells reached about 70–80% confluence, and imaged under a fluorescence microscope (Nikon Eclipse 80i, Japan). The images of four fields of view were acquired randomly. All experiments were repeated 3 times.

### Migration and invasion ability assay

Scratching wound healing assay: Renal cancer cells were seeded in micro-insert 4-wells (ibidi GmbH, Germany). Since the cells reached 80% to 90% confluence, the inserts were withdrawn and 500 μm scratches were formed. The complete medium was then replaced with fresh medium without serum. Scratch images were obtained by microscopy (Olympus, Japan) at 0, 24, and 48 h after scratch formation.

Transwell assay: Renal cancer cells were cultured in an environment without serum for 24 h. In 200 μl of serum-free medium, 5 × 10^4^ cells were suspended and put into Matrigel-coated transwell chambers. The chambers were slowly placed into the lower chamber with 500 μl of 10% FBS medium. Then, the transwell chambers were withdrawn from the incubator after roughly 48 hours, the cells that had not broken through the membrane were removed using cotton swabs, and the cells that had been fixed with 4% methanol. A 1% crystal violet solution was used to stain the cells. Images of four fields of view were obtained randomly under the microscope. All experiments were repeated three times.

### Statistical analysis

All bioinformatics data were statistically analyzed using R software (version 4.2.1). Experimental images were processed and quantified using ImageJ software. The quantitative experimental data were analyzed by *T*-test to calculate the significance of the differences. Prognosis differences were assessed using the log-rank test. The association between factors and patient prognoses was estimated using the univariate/multivariate Cox regression technique. The level of correlation between the two groups was computed using the Pearson method. Data were presented as means ± SD. A statistical *P*-value < 0.05 was considered significant. ^*^p < 0.05, ^**^p < 0.01, ^***^p < 0.001.

### Data availability statement

All datasets generated for our research are introduced in the article.

## Supplementary Materials

Supplementary Figures

Supplementary Table 1

Supplementary Table 2

Supplementary Tables 3 and 4
