# Supplementary material for: Comprehensive analysis of disulfidptosis-related genes: a prognosis model construction and tumor microenvironment characterization in clear cell renal cell carcinoma
Source: Aging (Albany NY). 2024 Feb 14;16(4):3647–73. doi: 10.18632/aging.205550 (PMC10929811; doi:10.18632/aging.205550)
Supplement: Supplementary Figures [file aging-16-205550-s001.pdf]

SUPPLEMENTARY FIGURES

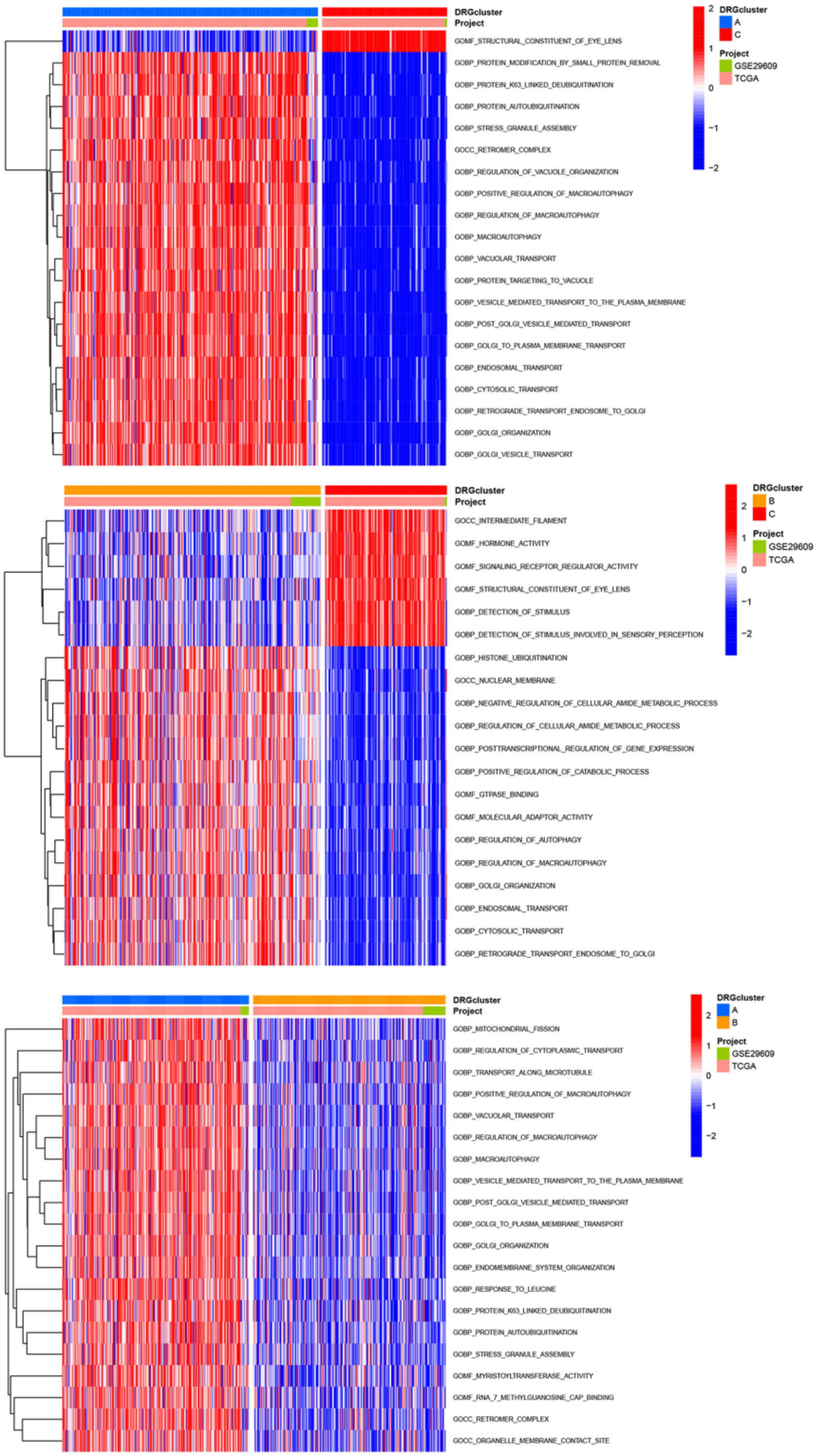

Supplementary Figure 1. GO GSVA analysis between DRG-related molecular subtypes.

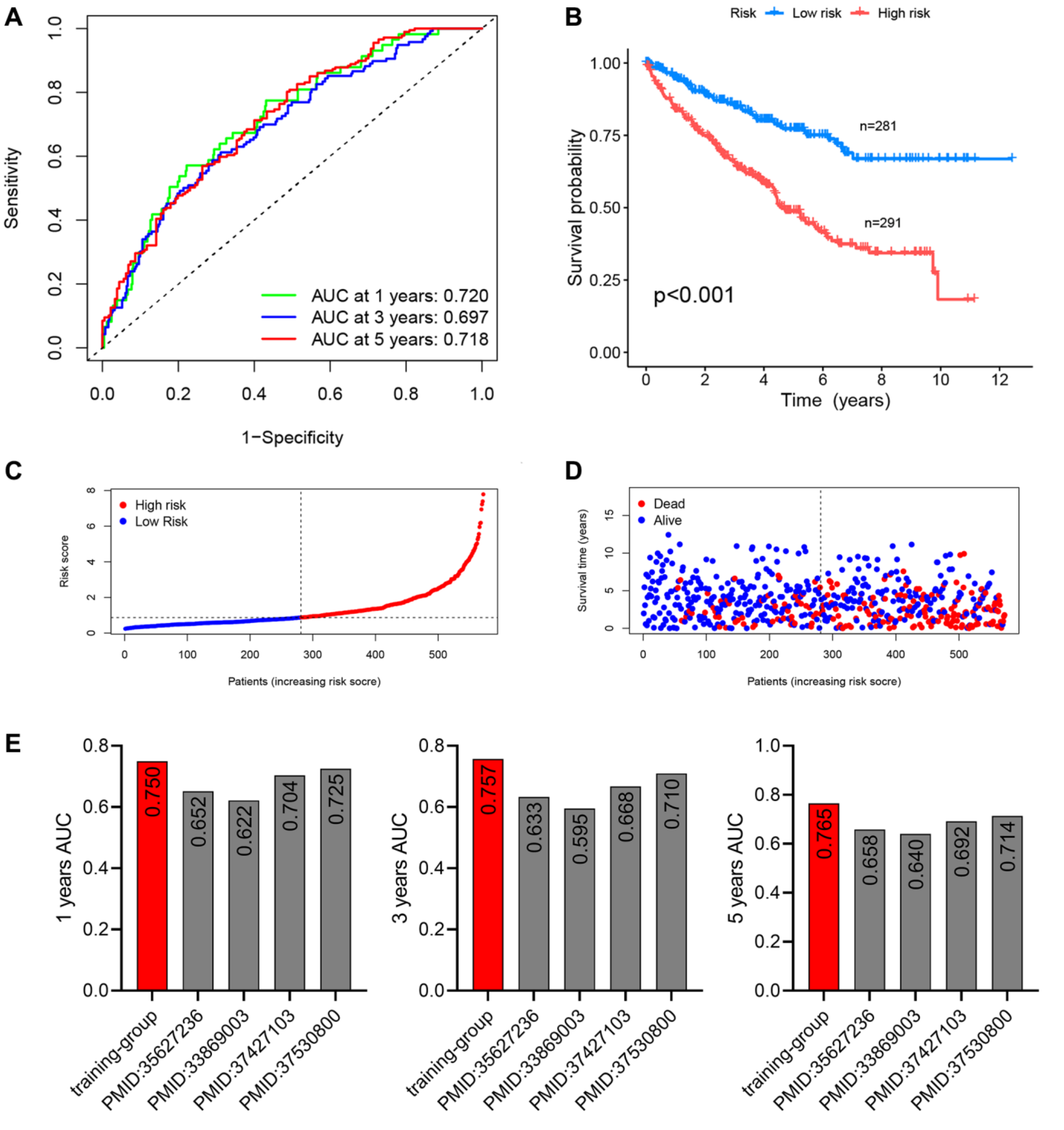

Supplementary Figure 2. (**A**–**D**) The ROC curves, KM curves, and point and scatter plots of risk score distribution and patient survival for the merged group. (**E**) Comparison of the DRG risk scoring model with other published ccRCC prognostic models.

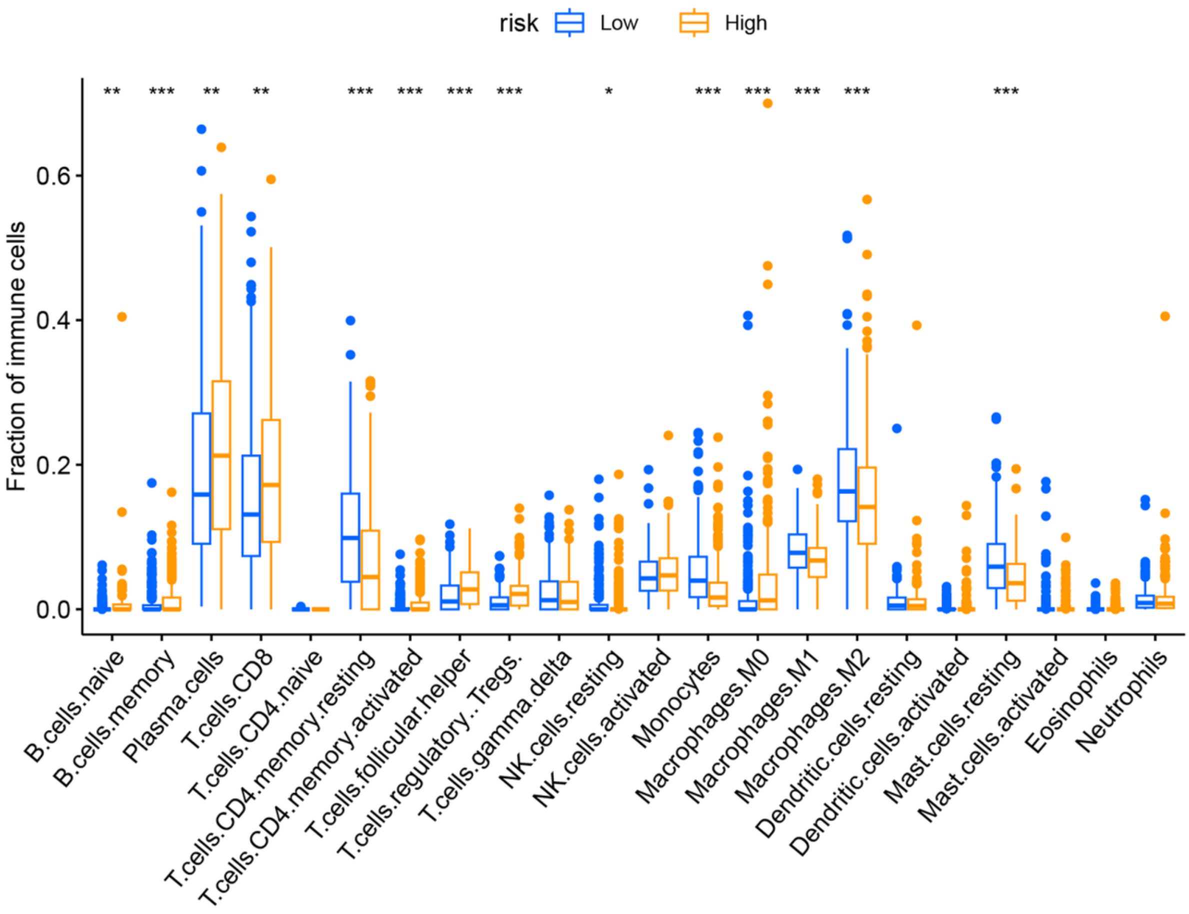

Supplementary Figure 3. Analysis of the percentage of immune cells in high and low-risk groups.

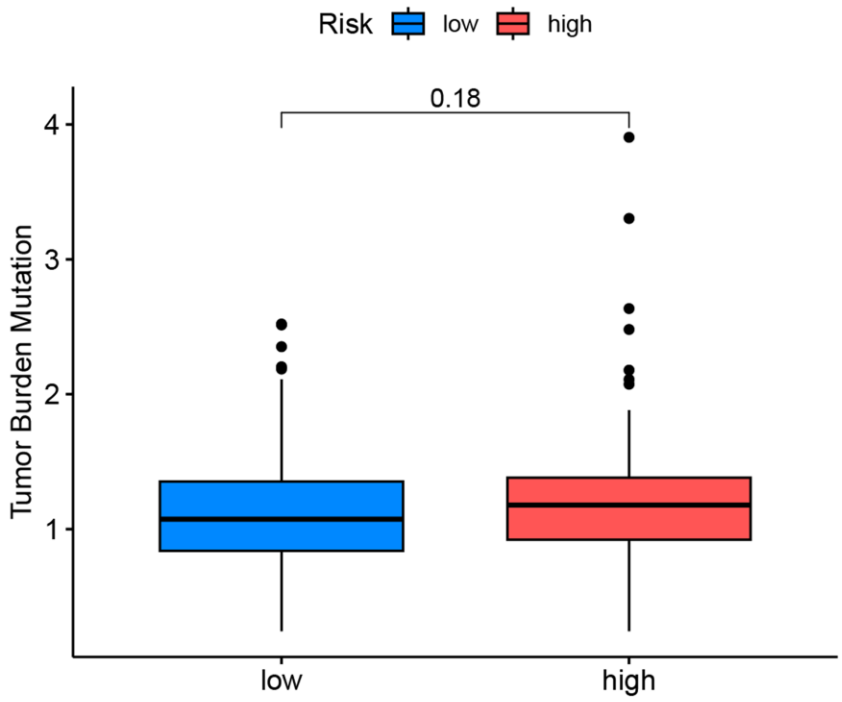

Supplementary Figure 4. TMB analysis of high and low-risk groups.

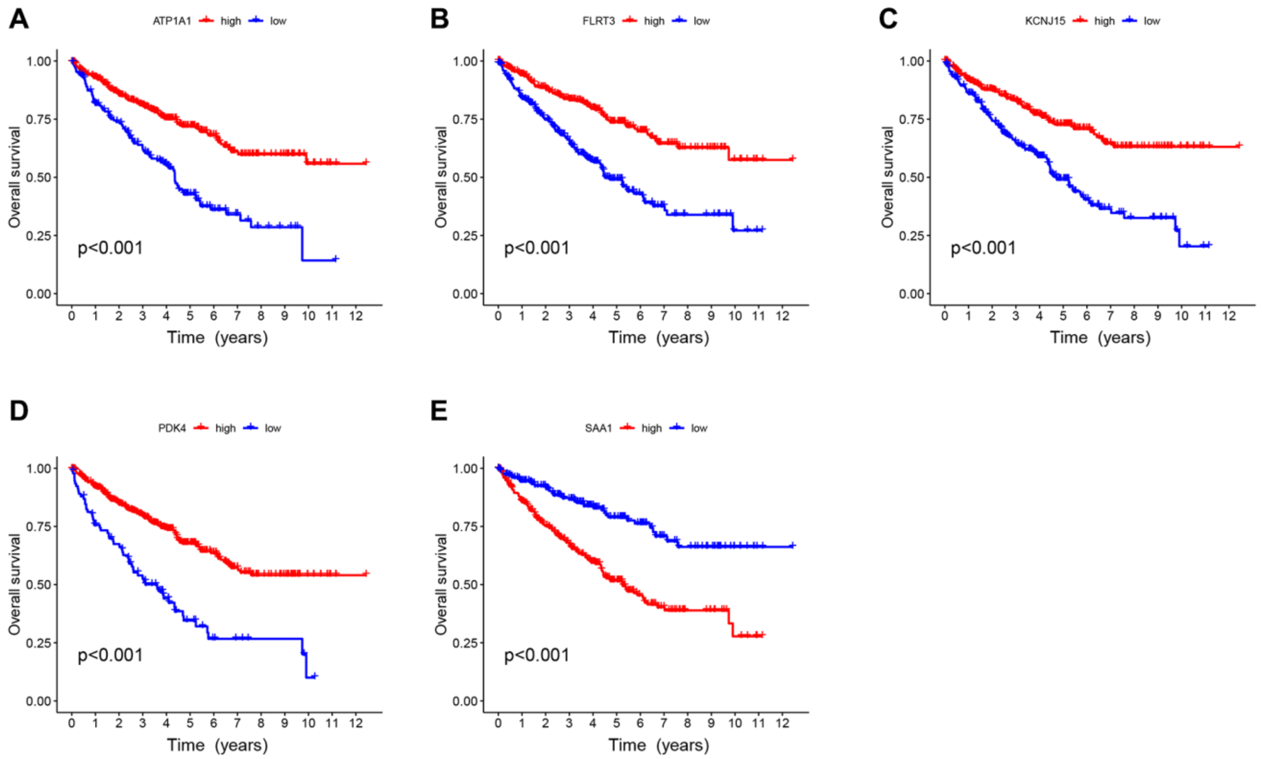

Supplementary Figure 5. Overall survival analysis of risk scoring genes in ccRCC. Includes (**A**) ATP1A1, (**B**) FLRT3, (**C**) KCNJ15 (**D**) PDK4, and (**E**) SAA1.
