# Supplementary material for: Comprehensive analysis of disulfidptosis-related genes: a prognosis model construction and tumor microenvironment characterization in clear cell renal cell carcinoma
Source: Aging (Albany NY). 2024 Feb 14;16(4):3647–73. doi: 10.18632/aging.205550 (PMC10929811; doi:10.18632/aging.205550)
Supplement: Supplementary Tables 3 and 4 [file aging-16-205550-s004.pdf]

SUPPLEMENTARY TABLES

Supplementary Table 3. Disulfidptosis related genes.

Gene	Type
GYS1	disulfidptosis
LRPPRC	disulfidptosis
NCKAP1	disulfidptosis
NDUFA11	disulfidptosis
NDUFS1	disulfidptosis
NUBPL	disulfidptosis
OXSM	disulfidptosis
RPN1	disulfidptosis
SLC3A2	disulfidptosis
SLC7A11	disulfidptosis

Supplementary Table 4. Risk score genes and their coefficients.

Id	coef
FLRT3	−0.113081065
ATP1A1	−0.188851917
SAA1	0.048476033
PDK4	−0.175069518
KCNJ15	−0.11452586
